# Supplementary material for: A Preliminary Study of the Potential Molecular Mechanisms of Individual Growth and Rumen Development in Calves with Different Feeding Patterns
Source: Microorganisms. 2023 Sep 28;11(10):2423. doi: 10.3390/microorganisms11102423 (PMC10609084; doi:10.3390/microorganisms11102423)
Supplement: Supplementary file 1 [file microorganisms-11-02423-s001.zip › Supplementary_Material.pdf]

Supplementary Material

A Preliminary Study of the Potential Molecular Mechanisms of Individual Growth and Rumen Development in Calves with Different Feeding Patterns

Jie Wang [1,†], Kaisen Zhao [2,†], Mianying Li [2], Huimei Fan [2], Meigui Wang [2], Siqi Xia [2], Yang Chen [2], Xue Bai [2], Zheliang Liu [2], Jiale Ni [2], Wenqiang Sun [1], Xianbo Jia [1] and Songjia Lai [1,*]

1 Farm Animal Genetic Resources Exploration and Innovation Key Laboratory of Sichuan Province, Sichuan Agricultural University, Chengdu 611130, China
2 College of Animal Science and Technology, Sichuan Agricultural University, Chengdu 611130, China
* Correspondence: laisj5794@163.com
† These authors contributed equally to this work.

1 Supplementary Tables

Table S1. The ingredients in the diets and the nutritional composition.

Items	Ingredients, %	Nutritional Composition	Content
Corn	61.15	NE[1], Mcal/kg	8.49
Soybean meal	30.05	CP, %	17.73
Compound Vitamin	0.03	Ca, %	3.1
Trace mineral premix	0.4	Available phosphorus, %	0.79
CaHCO3	0.67	Lys, %	0.99
CaCO3	1.65		
50% Choline chloride	0.1		
Soybean oil	4.02		
L-Lys	0.11		
DL-Met	0.43		
Mineral additive	1.39		

Note: 1. NE= net energy. Net energy for lactation was calculated based on the National Research Council of America recommendations (NRC, 2001).

Table S2. Sequence data summary of rumen contents samples generated

Sample	Raw Data	Clean Data	Clean_Q20	Clean_Q30	Clean_GC (%)	Contigs	N50 (bp)	ORFs
GF1	12,681.26	12,675.25	97.86	93.72	41.02	94,736	4,057	233,554
GF2	11,804.04	11,793.51	97.84	93.84	47.87	124,937	3,238	302,049
GF3	12,188.86	12,178.60	98.11	94.59	49.14	118,560	3,640	280,074
GFF1	82,953,020	12,436.05	97.98	94.22	48.45	152,910	3,112	354,972
GFF2	82,942,752	12,433.19	98.02	94.33	49.52	161,352	3,094	369,332
GFF3	81,157,812	12,167.75	98.21	94.71	47.8	170,820	3,682	415,290
TMR1	12,473.38	12,468.11	98.02	94.22	47.25	253,114	2,735	565,828
TMR2	12,169.36	12,164.51	98.12	94.49	47.53	232,460	3,396	541,533
TMR3	12,527.50	12,522.95	97.98	94.11	45.95	228,029	3,825	555,741
Total	247,127,428.4	110,839.92	882.14	848.23	424.53	1,536,918	30,779	3,618,373
mean	27,458,603.16	12,315.55	98.02	94.25	47.17	170,769	3,420	402,041
SD	-	-	0.12	0.33	2.53	55767	417	125813
SEM	-	-	0.04	0.11	0.84	18589	139	41938

Table S3. Relative abundances of the top 30 components of the microbiota at the genus level

genus	GF			GFF			TMR			Mean	SEM
	GF1	GF2	GF3	GFF1	GFF2	GFF3	TMR1	TMR2	TMR3		
Prevotella	15.06	24.43	26.08	30.19	26.54	30.16	35.61	34.68	27.09	27.76	2.04
Clostridium	30.51	7.74	3.48	5.51	3.80	4.05	3.65	2.74	4.31	7.31	2.94
Bacteroides	1.50	2.40	1.94	2.79	1.94	2.16	2.68	3.53	3.96	2.55	0.26
Ruminococcus	0.45	0.91	1.91	1.59	1.16	4.53	3.48	4.30	2.60	2.33	0.50
Eubacterium	1.02	1.46	1.02	1.82	2.17	1.94	0.62	0.68	0.70	1.27	0.20
Olsenella	0.99	1.39	2.66	1.70	3.01	0.45	0.03	0.38	0.22	1.20	0.36
Butyrivibrio	0.65	1.17	1.11	0.69	1.14	0.81	2.26	0.70	1.18	1.08	0.17
Selenomonas	0.40	2.03	3.75	0.40	0.24	0.42	0.29	0.92	0.43	0.99	0.39
Alistipes	0.27	0.49	0.32	1.83	0.45	0.70	1.14	1.82	1.05	0.90	0.20
Roseburia	0.31	1.23	0.28	1.05	0.61	0.60	0.22	0.90	1.84	0.78	0.18
Methanobrevibacter	1.11	1.00	0.67	0.41	2.09	0.75	0.16	0.26	0.44	0.77	0.20
Dialister	1.80	1.02	2.16	0.05	0.32	0.33	0.03	0.01	0.01	0.64	0.28
Mycoplasma	0.72	0.69	0.15	0.89	0.34	0.44	0.17	0.42	0.26	0.45	0.09
Succiniclasticum	0.07	0.60	0.17	0.51	0.97	0.41	0.96	0.16	0.16	0.45	0.12
Oribacterium	0.57	0.48	0.36	0.46	0.75	0.25	0.13	0.36	0.30	0.41	0.06
Treponema	0.06	0.05	0.03	0.12	0.11	0.32	0.97	0.94	1.04	0.40	0.15
Lachnoclostridium	0.43	0.40	0.45	0.35	0.54	0.33	0.20	0.25	0.30	0.36	0.03
Fibrobacter	0.05	0.09	0.09	0.12	0.38	0.19	0.40	0.92	0.70	0.33	0.10
Acidaminococcus	0.53	0.40	0.79	0.31	0.56	0.18	0.08	0.02	0.02	0.32	0.09
Megasphaera	0.20	0.28	1.60	0.12	0.25	0.16	0.05	0.05	0.02	0.30	0.17
Sharpea	0.22	0.61	1.09	0.11	0.12	0.23	0.02	0.23	0.02	0.29	0.12
Pseudobutyrivibrio	0.14	0.23	0.16	0.22	0.11	0.13	0.83	0.26	0.40	0.28	0.07
Blautia	0.39	0.28	0.29	0.26	0.36	0.25	0.13	0.24	0.17	0.26	0.03
Chlamydia	0.45	0.26	0.26	0.22	0.18	0.10	0.18	0.11	0.28	0.23	0.03
Succinatimonas	0.01	0.02	0.32	0.15	0.07	0.28	0.11	0.10	0.81	0.21	0.08
Sarcina	0.15	0.13	0.22	0.11	0.26	0.10	0.09	0.41	0.20	0.19	0.03
Paraprevotella	0.04	0.06	0.05	0.13	0.03	0.07	0.51	0.27	0.46	0.18	0.06
Parabacteroides	0.11	0.16	0.13	0.23	0.15	0.15	0.17	0.23	0.22	0.17	0.01
Desulfovibrio	0.21	0.22	0.28	0.19	0.26	0.08	0.06	0.04	0.04	0.15	0.03
Mitsuokella	0.17	0.33	0.36	0.12	0.13	0.10	0.03	0.04	0.06	0.15	0.04

Supplementary table 4 and supplementary table 5 see separate excel file.

Table S6. Significant metabolites between the GF and TMR groups,

Metabolite	VIP	FC	P value	Trend
**Epitestosterone**	0.085176	-3.55341	1.53E-13	down
**Androsterone**	0.005471	-7.51406	5.37E-08	down
**Palmitoleic Acid**	0.087759	-3.51032	2.41E-07	down
**11-Oxoetiocholanolone**	4.461098	2.157399	9.64E-07	up
**2,6-Di-tert-butyl-1,4-benzoquinone**	5.418781	2.437968	2.88E-06	up
**9,10-Dihome**	0.220619	-2.18037	3.89E-06	down
**Palmitoylcarnitine**	0.351551	-1.50819	5.72E-06	down
**Ursodeoxycholic acid**	6.908128	2.788295	9.51E-06	up
**Hexanoylcarnitine**	0.502094	-0.99397	1.31E-05	down
**7-Ketolithocholic acid**	5.831286	2.543814	2.03E-05	up
**Beta-Muricholic acid**	6.183549	2.628435	2.92E-05	up
**Heptadecanoic Acid**	0.192555	-2.37666	4.65E-05	down
**Testosterone**	3.581089	1.840399	5.19E-05	up
**Tiglic acid**	2.08895	1.062778	5.26E-05	up
**Propionylcarnitine**	1.827333	0.869739	9.48E-05	up
**Decanoylcarnitine**	0.345322	-1.53399	0.001585	down
**Vitamin A**	1.61797	0.694185	0.00276	up
**2-(14,15-Epoxyeicosatrienoyl) glycerol**	1.922119	0.942697	0.004456	up
**Taurocholic acid**	0.349857	-1.51516	0.007222	down
**Acetyl-L-carnitine**	0.608588	-0.71646	0.021674	down
**Taurodeoxycholic Acid**	0.292342	-1.77427	0.033304	down
**4-Methylvaleric Acid**	0.221152	-2.17689	0.037579	down
Styrene	4.30285	2.105292	1.28E-06	up
o-Cresol	0.541454	-0.88509	4.18E-05	down
Paracetamol	0.211056	-2.2443	0.000201	down
Riboflavin-5-phosphate	6.356555	2.668245	6.12E-05	up
dopaquinone	0.117704	-3.08677	9.1E-11	down
N-Propionylglycine	5.463866	2.449922	1.15E-07	up
Pipecolic acid	0.072307	-3.78973	4.16E-06	down
Glu-Gln	1.720138	0.782525	3.56E-05	up
DL-Serine	1.638128	0.712048	0.00035	up
L-Argininosuccinate	0.257499	-1.95736	0.002327	down
Isovalerylglycine	0.605081	-0.7248	0.009756	down
1-Methylhistidine	0.624108	-0.68013	0.013888	down
L-Carnitine	0.220993	-2.17792	0.012197	down
Sedanolide	5.311835	2.40921	2.88E-06	up
Isoquinoline	0.530213	-0.91536	0.000264	down
Methyl indole-3-acetate	0.541723	-0.88437	0.002006	down
3-Indoleacrylic acid	0.595377	-0.74812	0.008792	down
Indole-3-lactic acid	0.543787	-0.87889	0.010346	down
Indole-3-acetic acid	0.12784	-2.96759	0.019398	down
2-Hydroxy-6-Aminopurine	2.103167	1.072564	0.022147	up
5-Hydroxytryptophol	0.597419	-0.74319	0.026102	down
Isorhapontigenin	37.07851	5.212511	7.98E-07	up
13,14-Dihydro prostaglandin E1	0.058847	-4.0869	6.27E-11	down
N-(4-butyl-2-methylphenyl)-N'-[4-(4-methylpiperazino) phenyl] urea	0.021969	-5.50837	4.39E-10	down
Ergosta-5,7,9(11),22-Tetraen-3-beta-Ol	0.025693	-5.28246	4.4E-10	down
tetranor-12(R)-HETE	0.127344	-2.97319	2.03E-09	down
ethyl 2,3-dioxo-1,2,3,4-tetrahydroquinoline-4-carboxylate	0.287788	-1.79692	5.69E-09	down
4-methoxy-6-[2-(4-methoxyphenyl) ethyl]-2H-pyran-2-one	10.37472	3.375001	1.29E-08	up
4-(2,4-dichlorophenoxy)-3-methyl-4,5-dihydro-1H-pyrazol-5-one	4.24513	2.085809	1.7E-08	up
3-(methylsulfonyl)-2H-chromen-2-one	2.934822	1.553273	8.41E-08	up
L-Methionine sulfone	0.150599	-2.73122	1.01E-07	down
AL 8810 Methyl ester	0.077726	-3.68546	1.24E-07	down
jwh-018-d11	12.09775	3.596667	1.29E-07	up
1,4-dihydroxyheptadec-16-en-2-yl acetate	0.218598	-2.19365	3.43E-07	down
2,4-dihydroxyheptadec-16-en-1-yl acetate	0.023661	-5.40132	3.49E-07	down
Meperidine-d5	0.222874	-2.1657	1.11E-06	down
Glycocholic acid hydrate	5.395115	2.431654	3.05E-06	up
3-(3,4-dimethoxyphenyl)-1-(2-hydroxy-4,6-dimethoxyphenyl) propan-1-one	12.13587	3.601206	4.81E-06	up
ACar 18:0	0.299038	-1.7416	5.9E-06	down
2-(4,4-diphenyl-1-piperidinobuta-1,3-dienyl) phenyl acetate	0.204977	-2.28646	6.77E-06	down
4-Hydroxyisoleucine	0.11973	-3.06215	1.33E-05	down
13,14-dihydro-15-keto-tetranor Prostaglandin E2	0.559612	-0.8375	1.34E-05	down
LPC 18:3	0.406189	-1.29978	1.44E-05	down
DL-Stachydrine	0.104494	-3.25851	3.63E-05	down
D-Sphingosine	1.519783	0.603866	4.44E-05	up
Chenodeoxycholic acid-3-beta-D-glucuronide	9.544663	3.254694	6.29E-05	up
Dehydroepiandrosterone (DHEA)	0.153258	-2.70597	8.98E-05	down
8,8-dimethyl-2-phenyl-4H,8H-pyrano[2,3-h] chromen-4-one	0.052935	-4.23962	9.11E-05	down
Tetranor-12(S)-HETE	0.08113	-3.62363	9.38E-05	down
ACar 18:2	2.021728	1.015589	0.000109	up
ACar 13:0	0.25875	-1.95037	0.000129	down
(±)13-HpODE	0.074283	-3.75082	0.000183	down
11-Deoxy prostaglandin F1α	1.773922	0.826943	0.000207	up
Indole-3-acrylic acid	0.620251	-0.68908	0.000212	down
RKK	0.193961	-2.36616	0.000247	down
LPC 12:0	0.427646	-1.22551	0.000273	down
PC (14:1e/3:0)	0.554313	-0.85123	0.000282	down

Continued Table S6

(3R)-8-hydroxy-3-(4-methoxyphenyl)-3,4-dihydro-1H-2-benzopyran-1-one	2.983047	1.576787	0.000362	up
5α-Pregnan-3,20-dione	0.378051	-1.40335	0.000424	down
PC (9:0/9:0)	0.387074	-1.36932	0.00048	down
Clinafloxacin	0.3969	-1.33315	0.000673	down
PC (18:5e/2:0)	0.555482	-0.84819	0.000735	down
6-(trifluoromethoxy) quinolin-4-ol	3.155749	1.657983	0.000752	up
15-Deoxy-Δ12,14-prostaglandin A1	2.390691	1.257428	0.000777	up
PC (15:0/15:1)	0.386191	-1.37261	0.000786	down
Furanyl fentanyl 3-furancarboxamide isomer-d5	0.17759	-2.49337	0.000799	down
SM (d27:0/12:1)	2.449932	1.292742	0.000946	up
SM (d14:1/24:0)	1.858405	0.894065	0.000979	up
ACar 17:0	0.52436	-0.93137	0.001178	down
LPC 14:1	3.315666	1.729299	0.001283	up
4-[3-(4-phenoxyphenyl)-1H-pyrazol-5-yl] morpholine	0.608236	-0.7173	0.002846	down
13,14-dihydro-15-keto Prostaglandin A2	1.543894	0.626574	0.003697	up
PC (14:1e/6:0)	2.146886	1.102245	0.003786	up
N1-pyrazin-2-yl-4-chlorobenzamide	2.617593	1.388241	0.003868	up
HKK	0.251042	-1.994	0.003949	down
SM (d15:0/20:1)	0.6047	-0.72571	0.006041	down
3-hydroxy-4-methoxy-9H-xanthen-9-one	1.947836	0.961872	0.00722	up
PE (18:2/18:2)	0.653109	-0.6146	0.007332	down
PC (16:1e/20:0)	1.530652	0.614146	0.007815	up
PC (20:1/20:2)	2.421835	1.2761	0.008209	up
PC (19:0/20:5)	1.537841	0.620906	0.008266	up
Sunitinib	0.22682	-2.14038	0.008876	down
Amitriptyline-d3	0.083886	-3.57543	0.009544	down
(4-benzhydrylpiperazino) (1,5-dimethyl-1H-pyrazol-3-yl) methanone	2.920673	1.546301	0.010929	up
α-Hydroxyhippuric acid	2.556141	1.353967	0.011896	up
N-acetyl-L-ornithine	0.551915	-0.85748	0.012115	down
PC (16:1e/18:1)	3.262512	1.705983	0.012411	up
SM (d14:0/18:0)	0.62309	-0.68249	0.01286	down
SM (d16:2/26:1)	2.746537	1.457614	0.013967	up
PC (14:0e/24:4)	1.651844	0.724078	0.015014	up
LPC 18:4	0.433158	-1.20703	0.018831	down
N1,N2-dicyclohexylethanedithioamide	0.318745	-1.64953	0.024201	down
ACar 14:1	0.478286	-1.06406	0.030068	down
D-Galactosamine	1.696959	0.762952	0.031878	up
Taurodeoxycholic Acid (sodium salt)	0.260553	-1.94035	0.03565	down
(5-methyl-3-isoxazolyl) [4-(5-propyl-2-pyrimidinyl) piperazino] methanone	0.50429	-0.98768	0.036917	down
PC (7:0/7:0)	0.262422	-1.93004	0.037419	down
PC (18:5e/22:5)	0.636896	-0.65087	0.041886	down
PC (22:6e/17:2)	1.588216	0.667407	0.049604	up
Note: Lipids and lipid-like molecule metabolites were presented in bold font, the following tables was the same				

Table S7. Significant metabolites between the GFF and TMR groups.

Metabolite	VIP	FC	P value	Trend
**Hexanoylcarnitine**	2.184614	0.451334	4.74E-06	down
**Palmitoleic Acid**	1.801714	0.165562	8.60E-06	down
**Heptadecanoic Acid**	1.974865	0.228594	6.60E-05	down
**9,10-Dihome**	1.326429	0.441777	0.000389	down
**Taurocholic acid**	2.030551	0.254374	0.002342	down
**Taurodeoxycholic Acid**	1.706132	0.259256	0.022785	down
**Palmitoylcarnitine**	1.029993	0.640432	0.023136	down
**Decanoylcarnitine**	1.151423	0.554707	0.028947	down
**16(R)-HETE**	1.337629	0.533966	0.043635	down
o-Cresol	1.652578	0.611953	0.002344	down
IPH	1.668042	2.310731	0.022805	up
N-Propionylglycine	1.650525	3.423688	0.000387	up
D-Ala-D-Ala	1.711712	0.542838	0.002973	down
dopaquinone	1.136196	0.431174	0.025038	down
L-Argininosuccinate	1.24615	0.417533	0.029247	down
L-Carnitine	1.862692	0.193792	0.008495	down
Linoleoyl ethanolamide	1.881713	2.269418	0.009554	up
3-Indoleacrylic acid	2.007874	0.481604	1.67E-05	down
Methyl indole-3-acetate	1.847491	0.441264	0.009371	down
Indole-3-acetic acid	1.584154	0.144116	0.023619	down
Triptolide	1.57445	0.554322	0.025197	down
2-Hydroxy-6-Aminopurine	1.312564	1.729546	0.045833	up
tetranor-12(R)-HETE	1.741384	0.230235	2.38E-09	down

Continued Table S7

AL 8810 Methyl ester	2.055217	0.103251	1.17E-07	down
PC (9:0/9:0)	2.152611	0.315063	2.66E-07	down
Tetranor-12(S)-HETE	1.597186	0.176019	8.81E-07	down
2,4-dihydroxyheptadec-16-en-1-yl acetate	1.621011	0.086297	1.17E-06	down
L-Methionine sulfone	1.676834	0.267246	2.02E-06	down
2-(4,4-diphenyl-1-piperidinobuta-1,3-dienyl) phenyl acetate	2.087339	0.197223	3.77E-06	down
13,14-Dihydro prostaglandin E1	1.93679	0.101897	6.66E-06	down
LPC 18:3	1.868604	0.461835	1.67E-05	down
4-oxododecanedioic acid	2.014745	0.542495	2.23E-05	down
11-Deoxy prostaglandin F1α	2.199988	2.086662	3.52E-05	up
N1-isopropyl-2-(1H-2-pyrrolylcarbonyl)-1-hydrazinecarboxamide	2.037487	2.893975	4.06E-05	up
Furanyl fentanyl 3-furancarboxamide isomer-d5	1.264298	0.334094	4.34E-05	down
ethyl 2,3-dioxo-1,2,3,4-tetrahydroquinoline-4-carboxylate	1.632857	0.435656	7.68E-05	down
8,8-dimethyl-2-phenyl-4H,8H-pyrano[2,3-h] chromen-4-one	1.899983	0.086515	8.51E-05	down
(4-benzhydrylpiperazino) (1,5-dimethyl-1H-pyrazol-3-yl) methanone	1.963733	3.802065	9.93E-05	up
Ergosta-5,7,9(11),22-Tetraen-3-beta-Ol	1.313671	0.160178	0.000163	down
1,4-dihydroxyheptadec-16-en-2-yl acetate	1.307535	0.437101	0.000188	down
PC (18:3e/4:0)	1.906969	2.103232	0.000392	up
Dehydroepiandrosterone (DHEA)	1.733032	0.228333	0.000409	down
PC (18:5e/2:0)	1.661298	0.612325	0.000758	down
Lysopc 20:0	1.788728	1.907782	0.000948	up
HKK	1.234948	0.401731	0.000961	down
6-(trifluoromethoxy) quinolin-4-ol	1.83321	3.484199	0.001084	up
D-Sphingosine	2.187678	1.935016	0.001142	up
SM (d15:0/20:1)	1.936465	0.505545	0.001202	down
(±)13-HpODE	1.350434	0.226567	0.001847	down
4-(2,4-dichlorophenoxy)-3-methyl-4,5-dihydro-1H-pyrazol-5-one	1.277807	2.262634	0.002313	up
INK	1.786121	1.569337	0.002472	up
ACar 13:0	1.305094	0.435202	0.002526	down
(3R)-8-hydroxy-3-(4-methoxyphenyl)-3,4-dihydro-1H-2-benzopyran-1-one	1.677895	2.660637	0.003081	up
4-(methylthio)-6-phenyl-2-(3-pyridyl) pyrimidine-5-carbonitrile	1.78169	1.69828	0.004933	up
PC (16:1e/20:3)	1.345674	0.629633	0.005749	down
PC (14:0e/22:3)	1.717246	0.649957	0.006108	down
PC (18:0/19:2)	1.705066	0.619451	0.006115	down
3-(3,4-dimethoxyphenyl)-1-(2-hydroxy-4,6-dimethoxyphenyl) propan-1-one	1.545547	7.050015	0.00639	up
PC (14:1e/6:0)	1.126292	1.524199	0.006632	up
SM (d14:0/18:0)	1.705606	0.58375	0.006893	down
RKK	1.235362	0.402108	0.007057	down
4-methoxy-6-[2-(4-methoxyphenyl) ethyl]-2H-pyran-2-one	1.633183	8.659611	0.009323	up
Sunitinib	1.71734	0.245646	0.009816	down
PC (16:0e/22:6)	1.801976	0.440002	0.010119	down
SM (d20:0/13:0)	1.748705	0.570485	0.010528	down
Amitriptyline-d3	1.790578	0.090096	0.011125	down
PC (16:1/17:2)	1.002683	0.594209	0.012043	down
SM (d21:0/13:0)	1.750354	0.557355	0.012558	down
PC (7:0/7:0)	1.828365	0.18807	0.01261	down
(5-methyl-3-isoxazolyl) [4-(5-propyl-2-pyrimidinyl) piperazino] methanone	1.378296	0.460715	0.014449	down
PC (18:4e/22:1)	1.593088	1.625932	0.017694	up
PC (18:4e/20:5)	1.186887	0.583671	0.018265	down
PC (18:1e/14:1)	1.743653	0.444642	0.01879	down
1,2-dihydroxyheptadec-16-yn-4-yl acetate	1.462036	0.437438	0.021213	down
LPC 18:4	1.55936	0.438814	0.022488	down
PC (18:1/19:1)	1.486235	0.464814	0.024403	down
N1-pyrazin-2-yl-4-chlorobenzamide	1.517956	2.971583	0.024424	up
Taurodeoxycholic Acid (sodium salt)	1.686343	0.231056	0.024876	down
PC (14:1e/18:1)	1.605943	0.450125	0.025513	down
LPC 14:1	1.15913	2.211597	0.028683	up
PC (17:0/17:0)	1.199725	0.583845	0.038217	down
(R)-Equol	1.252756	1.630599	0.04106	up
3-hydroxy-1,5-diphenylpentan-1-one	1.201564	1.621385	0.042734	up
N1, N2-dicyclohexylethanedithioamide	1.407661	0.376279	0.046002	down
PC (22:5e/15:0)	1.084553	0.604675	0.049438	down

Table S8. Significant metabolites between the GF and GFF groups

Metabolite	VIP	FC	P value	Trend
**9,10-Dihome**	0.49939	1.11E-06	1.237518	down
**Epitestosterone**	0.101378	2.4E-05	2.196503	down
**16(R)-HETE**	2.052444	0.000107	1.8419	up
**Androsterone**	0.022211	0.000122	1.746916	down
**Decanoylcarnitine**	0.62253	0.00024	1.098005	down
**2-(14,15-Epoxyeicosatrienoyl) glycerol**	2.182782	0.000423	2.036677	up
**11-Oxoetiocholanolone**	2.954958	0.00144	1.789422	up
**7-Ketolithocholic acid**	3.216349	0.002368	1.626097	up
**Beta-Muricholic acid**	3.286759	0.002859	1.609573	up
**4-Methylvaleric Acid**	0.118101	0.003659	1.784835	down
**Testosterone**	2.467005	0.008733	1.65533	up
**7-Ketocholesterol**	1.846675	0.009311	1.590228	up
**Palmitoylcarnitine**	0.548928	0.011997	1.266373	down
**2,6-Di-tert-butyl-1,4-benzoquinone**	2.208031	0.025848	1.304452	up
**Ursodeoxycholic acid**	2.387726	0.031994	1.286866	up
**Cortisol**	0.512398	0.043626	1.64993	down
Styrene	2.982887	0.00081	1.837469	up
Paracetamol	0.26801	0.001703	1.730864	down
IPH	0.485262	0.007603	1.212649	down
Riboflavin-5-phosphate	4.934086	0.000112	1.897424	up
Pipecolic acid	0.095405	7.56E-06	2.091484	down
Isovalerylglycine	0.533754	0.001451	1.986333	down
dopaquinone	0.272985	0.024893	1.25191	down
Quinoline	0.539119	0.003743	1.997524	down
Sedanolide	2.316502	0.022908	1.373867	up
Isorhapontigenin	32.15428	8.96E-09	2.294917	up
N-(4-butyl-2-methylphenyl)-N'-[4-(4-methylpiperazino) phenyl] urea	0.069548	7.28E-06	1.781566	down
Meperidine-d5	0.205577	7.66E-06	2.273387	down
N1-isopropyl-2-(1H-2-pyrrolylcarbonyl)-1-hydrazinecarboxamide	0.341079	1.25E-05	2.192112	down
jwh-018-d11	6.100581	1.34E-05	1.845698	up
4-Hydroxyisoleucine	0.159483	4.18E-05	1.99936	down
DL-Stachydrine	0.166593	5.78E-05	1.907919	down
ACar 18:2	1.962208	0.000139	2.070392	up
Ergosta-5,7,9(11),22-Tetraen-3-beta-Ol	0.160405	0.000163	1.269289	down
RKK	0.482361	0.000298	1.125209	down
PC (14:1e/3:0)	0.581198	0.000315	1.982744	down
1,4-dihydroxyheptadec-16-en-2-yl acetate	0.500109	0.000353	1.228937	down
1,2-dihydroxyheptadec-16-yn-4-yl acetate	2.439234	0.000493	1.881058	up
PC (19:0/20:5)	1.671035	0.000563	1.912392	up
LPC 12:0	0.516274	0.000848	1.715679	down
ACar 17:0	0.45687	0.001667	1.998885	down
(±)13-HpODE	0.327865	0.002056	1.062657	down
PC (16:1e/18:1)	5.510062	0.002221	2.195877	up
Erdosteine	0.487314	0.00241	1.992432	down
(±)8-HEPE	1.513505	0.00255	1.230816	up
ACar 18:0	0.40637	0.003209	1.617459	down
PC (14:0e/24:4)	1.864413	0.003231	1.804686	up
4-oxododecanedioic acid	1.75444	0.003595	1.886244	up
5α-Pregnan-3,20-dione	0.563946	0.003677	1.375978	down
PC (16:0/17:1)	1.818787	0.004162	1.598579	up
Lysopc 20:0	0.566145	0.004244	1.716184	down
PC (18:3e/4:0)	0.55753	0.00473	1.642509	down
4-(2,4-dichlorophenoxy)-3-methyl-4,5-dihydro-1H-pyrazol-5-one	1.87619	0.005054	1.182793	up
Glycocholic acid hydrate	2.721339	0.00566	1.534292	up
5-[(10Z)-14-(3,5-dihydroxyphenyl) tetradec-10-en-1-yl] benzene-1,3-diol	2.039489	0.005718	1.505598	up
PC (18:2e/14:0)	3.050719	0.006214	1.729327	up
PC (22:4e/15:1)	0.665534	0.007422	1.408452	down
Clinafloxacin	0.443982	0.00933	1.709541	down
Chenodeoxycholic acid-3-beta-D-glucuronide	3.1739	0.017328	1.410122	up
2-(2-amino-3-methylbutanamido)-3-phenylpropanoic acid	0.567384	0.017909	1.582536	down
PC (19:0/19:1)	3.250051	0.020164	1.840894	up
PC (18:1/19:1)	2.390321	0.020978	1.67834	up
15-Deoxy-Δ12,14-prostaglandin A1	1.870861	0.026338	1.461569	up
N-acetyl-L-ornithine	0.619215	0.026381	1.542524	down
SM (d19:2/22:1)	0.595185	0.027098	1.234971	down
PC (20:1/20:2)	2.049862	0.031871	1.470827	up
3-Methoxy prostaglandin F1α	1.73771	0.034671	1.337911	up
PC (22:4e/13:0)	1.551786	0.035912	1.573126	up
D-Galactosamine	1.727622	0.036999	1.404621	up
PC (18:1e/14:1)	1.926216	0.037587	1.541477	up
PC (17:0/17:0)	1.843595	0.038597	1.442385	up
5-Hydroxyindole	1.903974	0.039176	1.331326	up
Furanyl fentanyl 3-furancarboxamide isomer-d5	0.531559	0.041304	1.020093	down
3-hydroxy-4-methoxy-9H-xanthen-9-one	1.647401	0.045452	1.378778	up
PC (16:0/17:2)	0.641059	0.046926	1.404322	down
PC (16:2e/22:6)	2.888857	0.047417	1.367909	up
PC (18:2e/22:5)	1.741183	0.048018	1.461514	up

2. Supplementary Figures

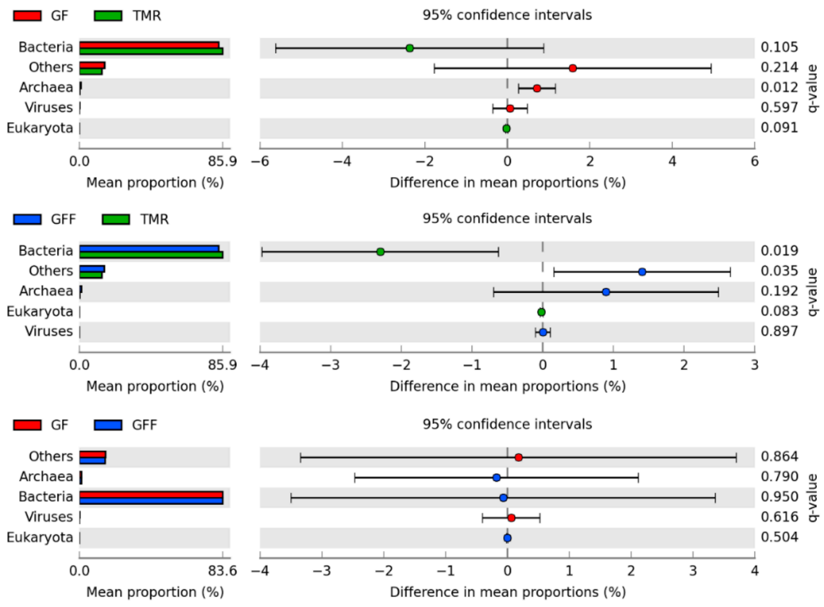

Supplementary Figure S1. Comparison of microbial domains of rumen in three groups.

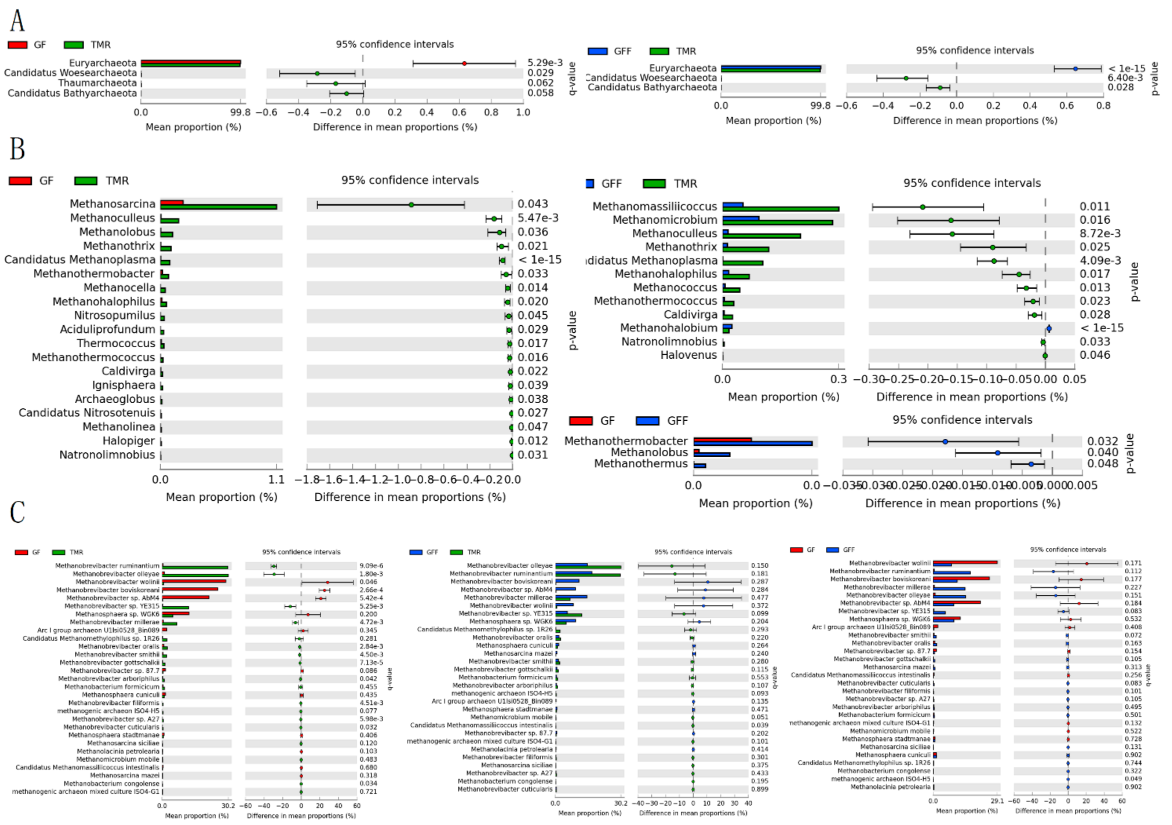

Supplementary Figure S2. Comparison of microbial in rumen archaea among the three groups. (A) Comparison of archaea at phylum level. (B) Comparison of archaea at the genus level. (C) Comparison of archaea at the species level.

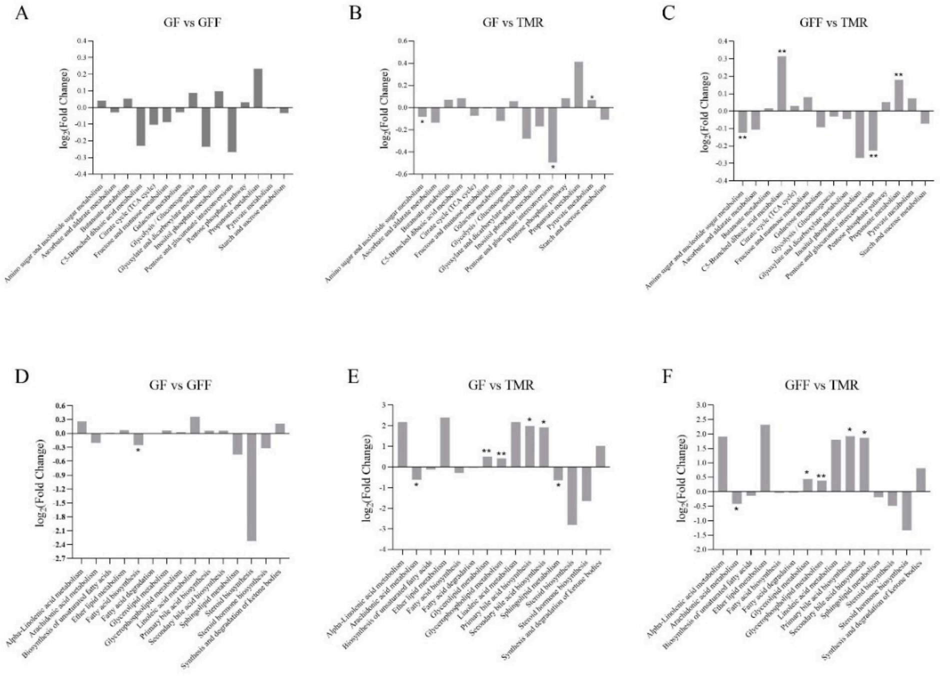

Supplementary Figure **S3.** Differential KEGG functions among the three groups. (A, B and C) Comparison of Carbohydrate metabolism of rumen microbial among the three groups. (D, E and F) Comparison of lipid metabolism of rumen microbial among the three groups. * *P*<0.05, significant difference, ** *P*<0.01, extremely significant difference.

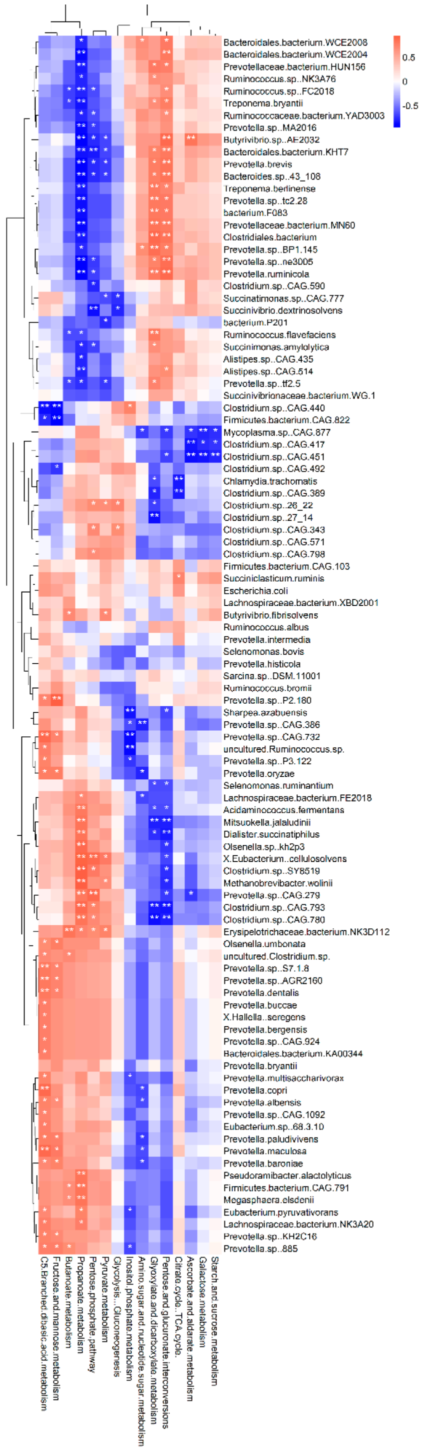

Supplementary Figure S4. Correlation analysis between top 100 microorganisms and carbohydrate metabolism pathways at species level

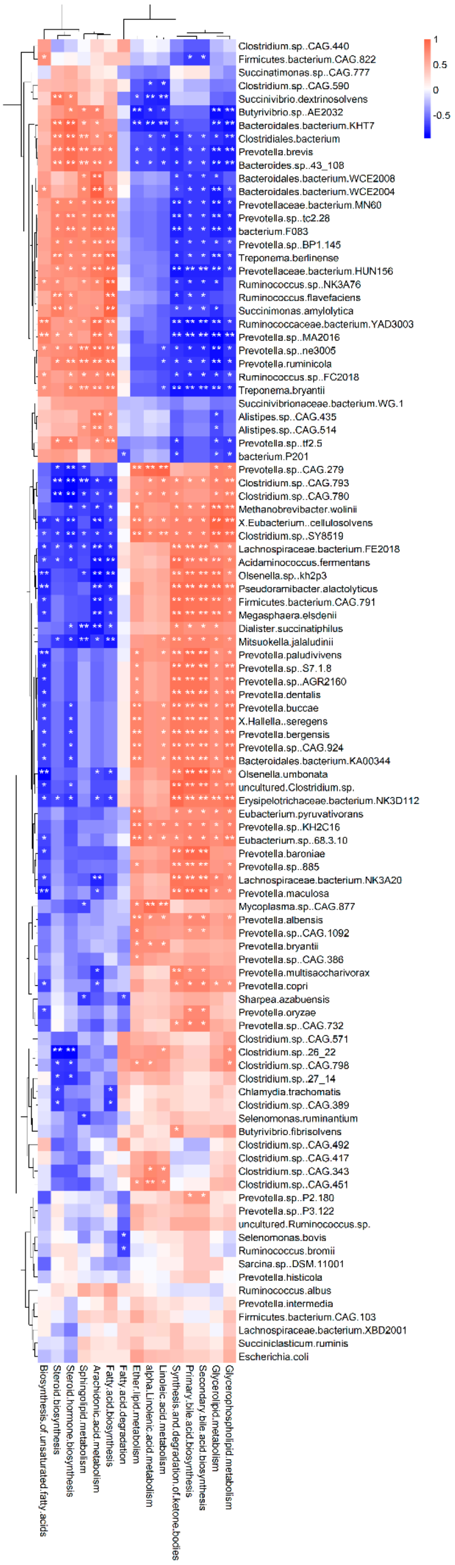

Supplementary Figure S5. Correlation analysis between top 100 microorganisms and lipid metabolism pathways at species level
